# Simultaneous Detection of Static and Dynamic Signals by a Flexible Sensor Based on 3D Graphene

**DOI:** 10.3390/s17051069

**Published:** 2017-05-08

**Authors:** Rongqing Xu, Di Wang, Hongchao Zhang, Na Xie, Shan Lu, Ke Qu

**Affiliations:** 1 College of Electronic Science and Engineering, Nanjing University of Posts and Telecommunications, Nanjing 210023, China; 1215022719@njupt.edu.cn (D.W.); xienns@njupt.edu.cn (N.X.); 2 National Laboratory of Solid-State Microstructures and Department of Materials Science and Engineering, Nanjing University, Nanjing 210093, China; 3 Department of Information Physics and Engineering, Nanjing University of Science and Technology, Nanjing 210094, China; hongchao@njust.edu.cn; 4 Shanghai Key Laboratory of Aerospace Intelligent Control Technology, Shanghai Aerospace Control Technology Institute, Shanghai 201109, China; 1215022718@njupt.edu.cn

**Keywords:** acoustic pressure sensor, three-dimensional graphene foam, flexible, wearable

## Abstract

A flexible acoustic pressure sensor was developed based on the change in electrical resistance of three-dimensional (3D) graphene change under the acoustic waves action. The sensor was constructed by 3D graphene foam (GF) wrapped in flexible polydimethylsiloxane (PDMS). Tuning forks and human physiological tests indicated that the acoustic pressure sensor can sensitively detect the deformation and the acoustic pressure in real time. The results are of significance to the development of graphene-based applications in the field of health monitoring, in vitro diagnostics, advanced therapies, and transient pressure detection.

## 1. Introduction

In recent years, flexible pressure sensors have been highly desirable in applications such as electronic skin, structural health monitoring, robot sensor, personal health monitoring, sport performance monitoring, and rehabilitation [1,2,3,4]. With the further development of robotics, biomechanics and medical measurement, the demand for flexible pressure sensor has become more urgent. Flexible pressure sensors are divided into two categories: conducting polymer sensors [5] and piezoelectric material sensors [6,7,8]. It is generally believed that conducting polymers as a stress sensor can only detect static stress, while piezoelectric sensors are only suitable for measuring dynamic behavior, such as vibration and acoustic waves. Currently, scientific breakthroughs are needed to simultaneously measure static and dynamic signals. Various flexible pressure sensors have been fabricated based on different nanomaterials, such as gold nanowire [9], ZnO nanowire [10], carbon nanotube (CNT) [4,11], and graphene [12]. Graphene in particular has sparked intensive interest for pressure sensors in the past few years owing to its superior mechanical and electrical properties [1,13,14]. Dong et al. reported highly sensitive electrochemical sensors for detection of [Fe(CN)_6_]^3+^ and dopamine with free-standing graphene/ZnO hybrid electrodes. Bae et al. reported a high-performance piezo-resistive pressure sensor device with a linear relationship between applied pressure and output, with a high sensitivity and a wide range of pressure by using two-dimensional (2D) monolayer graphene [15]. Recently, 3D graphene, such as graphene foams (GFs) [16], graphene sponges (GSs) [17], and graphene aerogels (GAs) [18], has attracted much more attention in a wide range of flexible sensors. Compared to monolayer graphene, the graphene sheets in GFs derived by template-directed chemical vapor deposition (CVD) are seamlessly interconnected to form a 3D flexible and free-standing graphene scaffolds. The unique interconnected network of GFs provides a great potential for use in composite materials for electrical applications. When the graphene composited with insulating polymer, the composites show good flexibility, and can be bent, stretched, and twisted without breaking. The high quality of the graphene sheets and their perfect connection in three dimensions provide the material with outstanding electrical and mechanical properties [19].

In our previous studies [20], only the static bending and tensile test signal is reported. The piezo-resistive stain sensors mostly detect signal by mechanical stimuli owing to its sensing mechanism based on the change in resistance due to mechanical deformation. Since the response time of the sensors can reach the second order for the large sample size and large deformation conditions, the sensors are suitable for the static stress measurement or low frequency signal measurement, such as muscle motions. None of the reported studies have claimed that this kind of sensor can measure dynamic signals of high frequency. This behavior can have an impact on the potential application of 3D graphene sensors. In this paper, our 3D GF sensors can detect the static signals from the mechanical deformation and the dynamic signals coupled by vibrations. Recently, it has been reported that suspended membrane configuration graphene sensors have orders of magnitude of higher sensitivity compared to other sensors [21]. Since 3D graphene has seamless interconnection and a free-standing scaffold, the GF sensors have high sensitivity. Here, we demonstrate a flexible acoustic pressure sensors based on 3D graphene network. A dynamic tuning forks acoustic signal was sensitively detected. Moreover, the physiological signals of human pulse and sound waves were captured to indicate an excellent response under static conditions. Particularly, the muscle motions and vocal cord vibrations during speech could both be detected by our sensor. We believe our work is of significance to graphene-based applications.

## 2. Materials and Methods

The 3D GF was synthesized by chemical vapor deposition method on nickel foam as a template. Ni foam was used to catalyze the graphene growth [20]. First, the nickel foam was cut to a desired size. It was sonicated in hydrochloric acid (HCl) solution, acetone, and deionized water, Second, the nickel foam was placed in the tube furnace and pre-treated in mixed gases with 25 SCCM hydrogen and 50 SCCM argon flow rates at 1000 °C for 10 min to eliminate a thin surface oxide layer. Subsequently, the carbon source of ethanol was introduced by a hydrogen and argon flow for 10 min to produce graphene. Finally, after cooling down the furnace naturally to room temperature under the protection of an argon flow, the graphene/nickel foam was corroded in a 10% HCl solution [22]. After that, the sample was cleaned three times by deionized water and dried at 60 °C.

**Figure 1:**
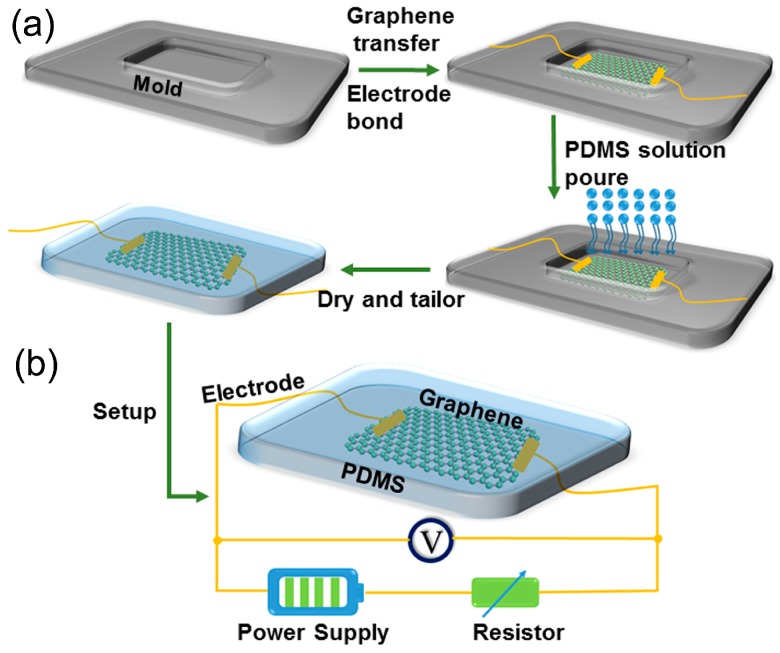
(**a**) Schematic illustration of overall fabrication process; (**b**) Schematic diagram of the experimental setup.

Figure 1a illustrates the fabrication process of our 3D graphene foam based flexible acoustic pressure sensor. First, a suitable mold was prepared for design 3D GF sensor. Then, the 3D GF was symmetrically bonded the silver plastic electrodes with two lead wires after being transferred in the mold. Subsequently, a small amount of PDMS solution (10:1 volume ratio pre-polymer and curing agent mixed) was poured into the mold. Finally, the sample was tailored to a desired size and shape to obtain a final 3D GF-based acoustic pressure sensor after the PDMS was dried at room temperature.

## 3. Results and Discussion

**Figure 2:**
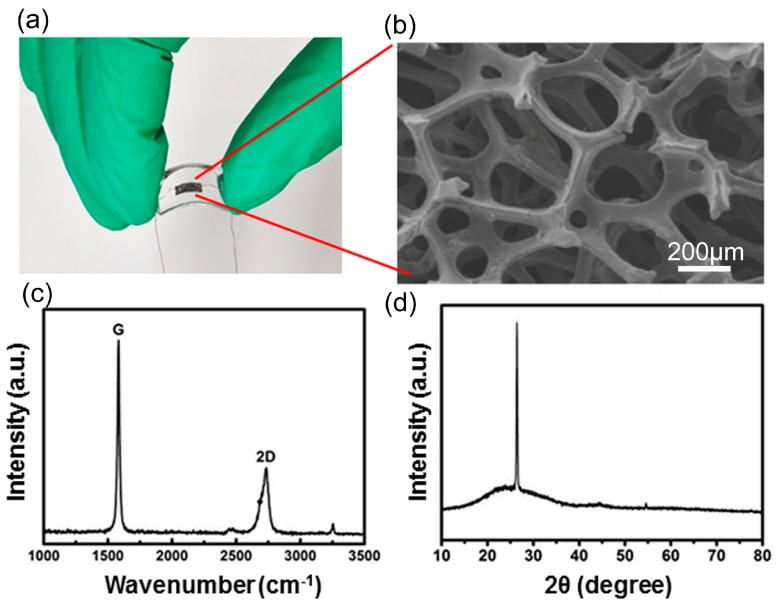
(**a**) Photograph of the acoustic pressure sensor; (**b**) SEM image of 3D graphene foam; (**c**) Raman spectrum of the 3D GF; (**d**) XRD patterns of the 3D GF.

Figure 2a shows the photograph of the acoustic pressure sensor. Such fabricated devices are wearable and bendable due to the flexible nature of both PDMS and graphene. Figure 2b presents the scanning electron microscopy (SEM) image of the 3D graphene foam, which shows the 3D GF exhibits a well-defined micro porous network structure with a pore diameter of about 200~400 μm.

The Raman spectrum of the 3D GF is shown in Figure 2c. The shape of the 2D band (~2700 cm^−1^) and the intensity ratio between the 2D and the G bands (~1560 cm^−1^) prove that the 3D GF is composed of few-layer or multilayer graphene sheets [23]. In addition, the Raman spectrum measured on free-standing GF shows a strongly suppressed defect-related D band (~1350 cm^−1^), indicating overall high quality of the graphene in GF. Figure 2d is an illustration of the X-ray diffraction (XRD) patterns of 3D GF. The intensity of the carbon peak in the pattern without nickel peak indicates a pure 3D pore structure of as-fabricated GF.

The size of the PDMS matrix for the 3D GF sensor used in the experiment is 30 × 10 × 5 mm^3^, the internal dimensions of the 3D GF composited in PDMS are 6 × 2 × 1 mm^3^. In order to study the frequency response characteristics of the sensor, we choose tuning forks as an excitation source. Figure 1b shows the tuning fork vibration setup. The sensor is attached at one arm of the tuning fork. The tuning forks begin to vibrate to generate a high frequency acoustic wave when the other arm is stroked with a hammer. This vibration signal with a high-frequency stress on GF, results in a mechanical deformation (compression and decompression) and recovery in GF. When the deformation and recovery occurs, the band gap of graphene dynamically changes, leading to the change of its resistance due to the piezo-resistive effect [24,25,26]. In our experiment, the acoustic wave propagation velocity in PDMS is over 1000 m/s. A voltage divider circuit is setup to evaluate the frequency signal detection capability. The fixed resistor (with resistance *R*_S_) and 3D GF sensor (with resistance *R*) are connected in series in the circuit. The voltage source is adjustable at 1~12 V. The voltage of the graphene sensor *V*_G_ is measured by an oscilloscope, where *R*_S_ is selected to satisfy *R*_S_ >> *R*. We usually take *R*_S_ ≈ 10 *R*_0_, and the output of the oscilloscope *V*_G_ is then related by

(1) $$V_{G}=\frac{R}{R_{S}+R}V_{S}\approx \frac{R}{R_{S}}V_{S}.$$

Here, *V*_S_ is the power supply voltage, while *R* and *R*_S_ are the resistances of the GF/PDMS sensor and the resistor, respectively, *R*_0_ refer to the initial resistance of GF/PDMS sensor without deformation. Equation (1) shows that the resistance of the GF sensor is proportional to the output voltage *V_G_* on the oscilloscope. This indicates the mechanical deformation and external stimuli can be detected by measuring the output voltage *V_G_*.

To carry out this test, five kinds of frequency tuning fork were chosen in the experiments, and their natural frequencies were calibrated with microphone. The natural frequency is, respectively, 128.0 Hz, 247.6 Hz, 516.8 Hz, 930.1 Hz, and 1873.0 Hz.

**Figure 3:**
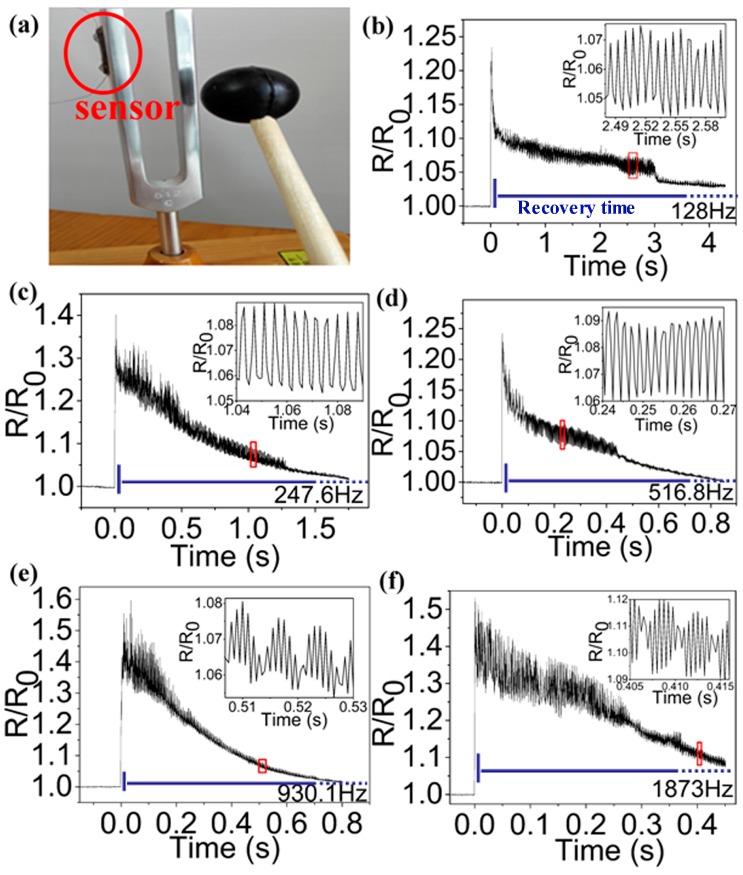
(**a**) A photograph of the experiment setup; (**b**–**f**) Signals obtained by the tuning forks with natural frequency, respectively: 128 Hz, 247.6 Hz, 516.8 Hz, 930.1 Hz, and 1873 Hz. The blue dotted line means recovery time should be longer than *x*-axis in (**b**–**f**). The insert is the high-frequency oscillation signal of the portion of the curve inside the red box.

Figure 3a is a photograph of the experiment setup to test the acoustic waves of the tuning forks. The sensor is adhered to one arm of the tuning fork. The tuning fork begins to vibrate when the other arm is stroked with a rubber hammer. The sensor is shaking, resulting in the dynamic variation on its resistance. Figure 3b–f show the variation of its resistance caused by the tuning fork vibration. The signals of Figure 3b–f were obtained by the tuning forks with natural frequency, respectively.

As shown in Figure 3b–f, the signals have instantaneous arising edge and then back up slowly. We believe that this phenomenon is caused by the suddenly mechanical deformation of the 3D GF due to the rubber hammer striking the tuning fork. When a rubber hammer instantaneously strikes the tuning fork, it causes a large amplitude mechanical deformation of the 3D GF momentarily, and induces a rapid change in the electrical resistance. As shown in Figure 3b–f, the steep rising edge is just a few milliseconds. It indicates that the 3D GF responses to mechanical deformation very quickly. However, the 3D GF has a relatively longer recovery time to the mechanical deformation. In Figure 3b–f, its recovery time even reached several seconds.

**Figure 4:**
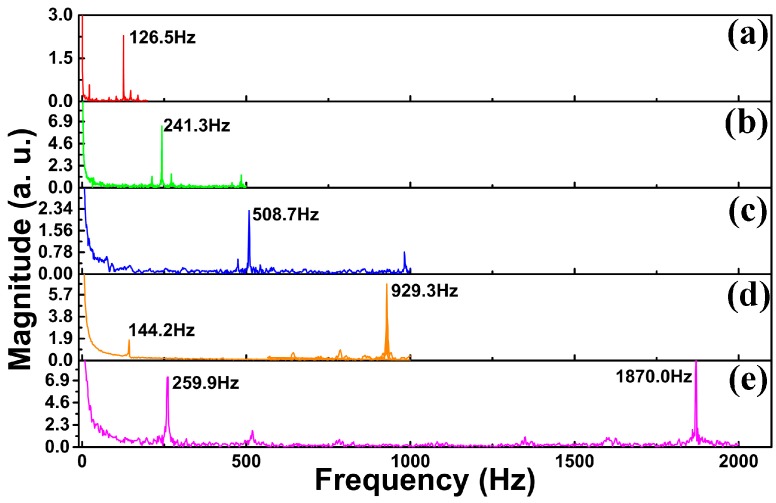
The spectra of insets of Figure 3b–f.

Figure 4 is the spectra of insets of Figure 3b–f with fast Fourier transform (FFT) algorithm. It shows that the five main frequencies of the acoustics are, respectively, 126.5 Hz, 241.3 Hz, 508.7 Hz, 929.3 Hz, and 1870 Hz. They are nearly equal to the natural frequency of the tuning fork. It indicates that the sensor can detect the natural frequency of the tuning fork. There is a small deviation between these two sets of values, because the frequency of the tuning fork is determined by the tuning fork structure, which changes when the sensor contacts the tuning fork, resulting in a small change in the frequency.

**Figure 5:**
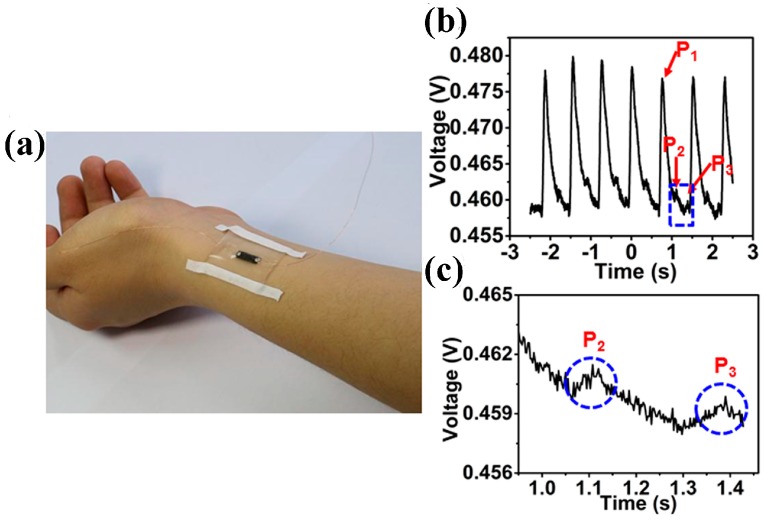
(**a**) Wrist pulse signal test with GF/PDMS sensor; (**b**) Typical radial artery pulse waveform obtained from GF/PDMS sensor; (**c**) Magnified peaks of P2 and P3 in (**b**).

The capability of monitoring human physiological signals is essential for 3D GF sensor to be applied in the fields of e-skin. A wrist pulse signal test with a 3D GF sensor is shown in Figure 5a. The wrist pulse signal was read out accurately as shown in Figure 5b, in which a typical radial artery pulse waveform was obtained with two clearly distinguishable peaks (P1 and P3) and a late systolic augmentation shoulder (P2). Figure 5c shows magnified peaks of P2 and P3 in Figure 5b. The line shape is known to be caused by the constitution of the blood pressure from the left ventricle contracts and a reflective wave from the lower body, and similar results were observed by Gong et al. [9].

**Figure 6:**
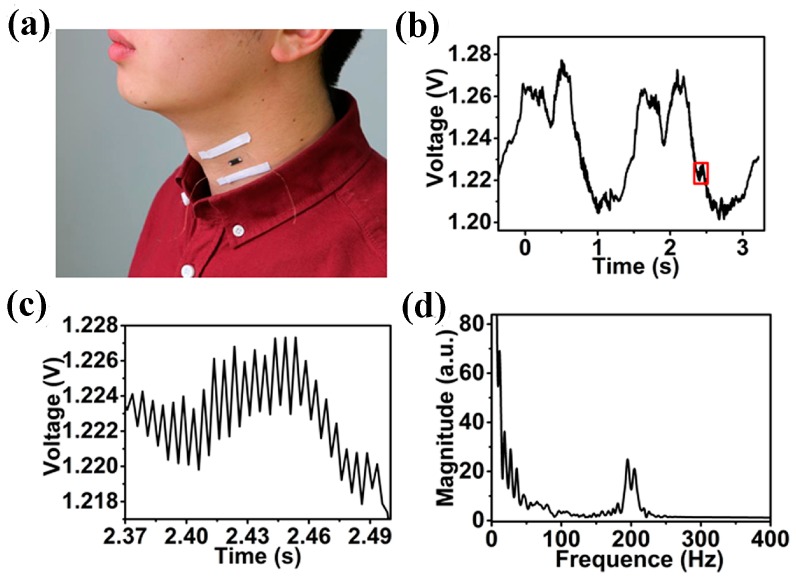
(**a**) Phonation signal test with GF/PDMS sensor; (**b**) The measured GF/PDMS sensor signal when a tester is speaking Chinese characters “Nanjing”; (**c**) The amplified view of high frequency oscillation signal in Figure 6b; (**d**) Fast Fourier transform of high frequency oscillation signal.

The flexible and highly sensitive 3D GF sensor was also attached to the skin of the neck for the phonetic signal test, as shown in Figure 6a. Figure 6b shows the measured 3D GF sensor signal of a tester speaking “Nanjing,” in which two distinguished waveforms could be ascribed to the change in resistance arising from muscle motions to pronounce individual syllables. Furthermore, high frequency signals superimposed on the waveforms are also observed (the labeled part in Figure 6b). Figure 6c shows the magnified view of high frequency signals, in which a regular oscillation signal is clearly observed. The characteristic frequency determined by fast Fourier transform (Figure 6d) is around 200 Hz, within the frequency range of human phonation.

## 4. Conclusions

We have developed an efficient, low-cost approach to fabricating a flexible and wearable acoustic pressure sensor based on 3D graphene foam. The sensor is made of flexible macro block material, easily made into various sizes and shapes, and it is economical, simple to prepare, easy to operate, easy to install and test, etc. More importantly, the test results show that the sensor has a good response to the natural frequency of the tuning fork, human pulse, and sound in real-time. Because of the distinctive features of high sensitivity and flexible, the sensor has a wide potential for health monitoring, in vitro diagnostics, advanced therapies, and transient pressure detection.
